# Protein Phosphatase 2A Deficiency in Macrophages Increases Foam Cell Formation and Accelerates Atherosclerotic Lesion Development

**DOI:** 10.3389/fcvm.2021.745009

**Published:** 2022-01-18

**Authors:** Rui Li, Chao Zhang, Fei Xie, Xianming Zhou, Xingjian Hu, Jiawei Shi, Xinling Du, Zhiyong Lin, Nianguo Dong

**Affiliations:** ^1^Department of Cardiovascular Surgery, Union Hospital, Tongji Medical College, Huazhong University of Science and Technology, Wuhan, China; ^2^Cardiology Division, Emory University School of Medicine, Atlanta, GA, United States

**Keywords:** PP2A (protein phosphatase 2A), atherosclerosis, foam cell formation, p38, CD36

## Abstract

Protein phosphatase 2A (PP2A), a crucial serine/threonine phosphatase, has recently been reported to play an important role in cardiovascular disease. Previous studies have hinted that PP2A is involved in atherosclerosis formation, but the associated mechanisms remain poorly understood. In this study, we investigate the role of PP2A in the pathogenesis of atherosclerosis. In human atherosclerotic coronary arteries, we found that the expression and activity of PP2A decreased significantly when compared to non-atherosclerotic arteries. Additional experiments demonstrated that pharmacological inhibition of PP2A aggravated atherosclerosis of ApoE^−/−^ mice. Considering the central role of macrophages in atherosclerosis, mice with conditional knockout of the PP2A-Cα subunit in myeloid cells were produced to investigate the function of PP2A in macrophages. Results showed that PP2A deficiency in myeloid cells aggravated atherosclerotic lesions in mice. *in vitro* experiments indicated that PP2A-deficient macrophages had an enhanced ability of lipid uptake and foam cell formation. Mechanistically, the deficiency of the PP2A in macrophages led to an increase in the phosphorylation level of p38, which contributed to the elevated expression of scavenger receptor CD36, a key factor involved in lipoprotein uptake. Our data suggest that PP2A participates in the pathophysiological process of atherosclerosis. The decrease of PP2A expression and activity in macrophages is a crucial determinant for foam cell formation and the initiation of atherosclerosis. Our study may provide a potential novel approach for the treatment of atherosclerosis.

## Introduction

Atherosclerosis is a chronic inflammatory disease associated with significant morbidity and mortality worldwide. It is characterized by the formation of fibrofatty lesions in the artery wall which can lead to many serious complications, such as myocardial infarction, stroke, and disabling peripheral artery diseases (1). Many cell types are involved in the development and progression of atherosclerosis, including vascular endothelial cells, fibroblasts, smooth muscle cells, and monocytes/macrophages (2, 3). Studies conducted over the past several decades have supported the concept that the initiation of atherosclerosis is the deposition and oxidative modification of low-density lipoprotein (LDL) in the intima of arteries. The uptake and treatment of oxidized LDL (oxLDL) by macrophages causes an overload of intracellular cholesterol esters leading to the formation of foam cells, a sign of early atherosclerotic lesion development (4, 5). However, despite decades of research, the molecular mechanisms that lead to foam cell formation in the process of atherosclerosis are still not fully understood.

Kinase and phosphatase mediated reversible protein phosphorylation plays a vital role in the regulation of many biological events (6). Protein phosphatase 2A (PP2A), a crucial serine/threonine phosphatase, consists of a scaffold subunit (PP2A-A), a regulatory subunit (PP2A-B) and a catalytic subunit (PP2A-C). Both PP2A-A and PP2A-C subunits have two isoforms, while the PP2A-B subunit has four different families (B, B′, B″, B^‴^) containing at least 13 different isoforms. This enables PP2A to fulfill a wide range of roles (7). The PP2A-C subunit has two types of post-translational modifications, phosphorylation at tyrosine-307 (Y307) and methylation at leucine-309 (L309). PP2A-C L309 methylation is essential for the maturation of PP2A and Y307 phosphorylation can result in decreased PP2A activity (8–10). Previous studies have shown that PP2A acts as a tumor suppressor in the development of many solid cancers and leukemias (11, 12). Emerging evidence suggests that PP2A activity also affects the pathogenesis in Alzheimer's disease and diabetes (13–15). In cardiovascular disease, PP2A activity is involved in a wide variety of pathophysiological processes, such as arrhythmia, cardiac remodeling, vascular remodeling, oxidative stress, ischemia-reperfusion injury, and endothelial dysfunction (16–20).

To our knowledge, there has been no direct research to investigate whether the expression and the activity of PP2A changes in atherosclerosis. In our study, using human coronary artery samples and mice with conditional knockout of the PP2A-Cα subunit in myeloid cells, we identified a role and associated molecular mechanisms of PP2A in foam cell formation and atherosclerosis.

## Materials and Methods

### Human Coronary Artery Collection

Human specimens used in this study were obtained from patients undergoing heart transplant previously diagnosed with coronary atherosclerosis by angiography. Both atherosclerotic coronary arteries and adjacent non-atherosclerotic coronary arteries were taken from the same patients. Specimens from each patient were divided into four parts according to the purpose of the experiment. Arteries snap-frozen and preserved in liquid nitrogen were used for western blot and PP2A activity assays. All of the human studies described in this work complied with the Declaration of Helsinki and were approved by the Ethics Committee of Union Hospital, Tongji Medical College, Huazhong University of Science and Technology (No: IORG0003571). Informed consent was signed by all patients.

### Animals

ApoE^−/−^ mice (C57BL/6 background) were purchased from Beijing HFK Bioscience Co., Ltd. Ppp2cα^flox/flox^ (fl/fl) and Cre^Lyz2^ mice (C57BL/6 background) were purchased from the Model Animal Research Center of Nanjing University. Mice were housed in a special pathogen free, temperature- and light-controlled animal facility at Tongji Medical College, Huazhong University of Science and Technology under a 12 h light/dark cycle and allowed *ad libitum* access to standard rodent chow and water. Ppp2cα^flox/flox^ and Cre^Lyz2^ mice were mated to produce Ppp2cα^flox/flox^ Cre^Lyz2^ (cKO) mice. For the pharmacological inhibition of the PP2A study, 10-week-old ApoE^−/−^ male mice were randomly divided into two groups: ApoE^−/−^ mice injected with LB100 (Selleck Chemicals, 1.0 mg/kg) and ApoE^−/−^ mice injected with normal saline (1.0 mg/kg). Both groups received intraperitoneal injections every 2 days and were fed a western diet (D12079B, ReadyDietech) for 12 weeks. For the AAV-PCSK9 injection model, 10-week-old fl/fl and cKO male mice were injected once with AAV-PCSK9 (1.0 × 10^11^ VG/mouse) or AAV-GFP (1.0 × 10^11^ VG/mouse) *via* tail vein and then fed with a high-fat diet (D12108C, ReadyDietech) containing 1.25% cholesterol for 16 weeks. AAV8-D377YmPCSK9 and AAV8-GFP were produced by Vigene Biosciences, Inc. When modeling was complete, mice were fasted for 12 h and exposed to 5% isoflurane inhalant with oxygen flow rate at 1 L/min. Then, the mice were monitored continuously during the procedure and the complete anesthesia was confirmed by a lack of pedal reflex. After the mice were completely anesthetized, blood was collected from the inferior vena cava and then centrifuged to obtain plasma. Total cholesterol and triglyceride levels were tested using commercial kits (MAK043 and MAK040, MERCK). The left ventricle was perfused with 20 ml saline containing heparin, and then the heart, aorta and liver were fixed in 4% paraformaldehyde or snap-frozen and preserved in liquid nitrogen. During this process, mice were euthanized due to exsanguination and thoracotomy. All animal procedures performed conformed to the current NIH Guide for the Care and Use of Laboratory Animals. All of the animal protocols were approved by the Institutional Animal Research Committee of Tongji Medical College, Huazhong University of Science and Technology (IACUC Number: 2439).

### PP2A Phosphatase Activity Assay

PP2A activity was measured using a commercial PP2A immunoprecipitation phosphatase assay kit (Millipore). Tissue or cell was lysed using a lysis buffer (20 mM imidazole-HCL, 2 mM EDTA, 2 mM EGTA, 1 mM benzamidine, pH 7.0 with 10 μg/ml aprotinin, and 1 mM PMSF) and the lysate was sonicated for 10 s and centrifuged at 2,000 *g* for 5 min. Supernatant was collected, total protein was quantified with a BCA Protein Assay kit (Beyotime Biotechnology), and 200 μg of total protein was incubated with anti-PP2A-C subunit antibody (4 μg) at 4°C for 18 h with gentle rocking. Then 40 μl Protein A agarose slurry was added and rocked at 4°C for 2 h. The mixture was centrifuged five times, during which beads were washed three times with 700 μl TBS and once with 500 μl Ser/Thr assay buffer. Then beads were incubated with 60 μl diluted phosphopeptide and 20 μl Ser/Thr assay buffer at 30°C for 10 min in a constant temperature shaker. Finally, the mixture was centrifuged briefly, and the supernatant was analyzed in a colorimetric assay using malachite green at an absorbance of 650 nm.

### Macrophage Isolation, Culture and Treatment

Eight- to Ten-week-old Ppp2cα^flox/flox^Cre^Lyz2^ and Ppp2cα^flox/flox^ male mice were injected with three milliliters of 3% thioglycolate. After 3 days, the mice were exposed to 5% isoflurane inhalant with oxygen flow rate at 1 L/min and were monitored continuously during this procedure. The complete anesthesia was confirmed by a lack of pedal reflex. After the mice were completely anesthetized, a well-trained researcher performed cervical dislocation to euthanize the mice in accordance with the current NIH Guide for the Care and Use of Laboratory Animals. Then, peritoneal macrophages were harvested by peritoneal lavage. Lavage fluid was centrifuged, and cells were re-suspended in RPMI 1640 medium (Gibco) containing 10% FBS (Gibco) and 1% penicillin-streptomycin (Gibco), plated onto 6-well plates (2 × 10^6^ cells per well) and then cultured in an incubator at 37°C with 5% CO_2_. After 2 h, non-adherent cells were removed, and macrophages were cultured in fresh RPMI 1,640 complete medium overnight. For foam cell formation assessment, cells were treated with oxidized low-density lipoprotein (ox-LDL; 50 μg/ml; Yiyuanbiotech) for 24 h. Cells were fixed with 4% paraformaldehyde and then stained by 0.5% Oil-Red-O (Sigma-Aldrich). For fluorescent labeling oxLDL (Dil-oxLDL, Yiyuanbiotech) uptake experiments, macrophages were incubated with Dil-oxLDL for 4 h. During the inhibitor tests, macrophages were treated with SB203580 (Selleck) or MK2206 (Selleck) for 4 h before ox-LDL treatment.

### Protein Extraction and Western Blot Analysis

Tissues or cells were lysed by RIPA buffer (Thermo Fisher) supplemented with protease and phosphatase inhibitors (Thermo Fisher). Lysates were sonicated and then cleared by centrifugation at 12,000 *g* for 15 min. Protein concentrations were quantified with a BCA Protein Assay kit (Beyotime Biotechnology). Equal amounts of proteins were separated by SurePAGE^TM^ precast polyacrylamide gels with a gradient between 4 and 20% (GenScript) and blotted onto 0.22 μm PVDF membranes (Millipore) by eBlot L1 Fast Wet Transfer System (GenScript). After blocking in TBST containing 5% skim milk for 1 h at room temperature, membranes were incubated with appropriate primary antibodies overnight at 4°C on a shaker. Specific banding was detected by incubation with appropriate HRP-conjugated secondary antibodies and an enhanced chemiluminescence (ECL) system (Thermo). Band intensities were quantified using ImageJ software. Detailed antibody information is provided in Supplementary Table 1.

### RNA Extraction and Quantitative RT-PCR Analysis

Total RNA was isolated from cells by Trizol reagent (Thermo Fisher) according to the protocol provided by manufacturer and then reverse transcribed into cDNA. The resulting cDNA was used as the template for quantitative RT-PCR. The reaction of quantitative RT-PCR was performed on a StepOne Plus thermal cycler (Applied Biosystems) according to the manufacturer's instructions. Detailed information of primers used in this study is listed in Supplementary Table 2. Gene expression was standardized to GAPDH using the 2^−Δ*Δct*^ method with each sample analyzed in triplicate.

### Atherosclerotic Lesion Analysis

After 48 h of fixation in 4% paraformaldehyde, aortas of mice were cut open longitudinally, stained with Oil-Red-O (Sigma-Aldrich) for 15 min, fixed on black paraffin wax dishes with insect mounting pins, and photographed. Digital images of Oil-Red-O stained aortas were quantified using ImageJ software.

### Histology and Immunohistochemistry

Cryosections (8 μm) from mouse aortic sinus were prepared, Hematoxylin and Eosin (H&E), Oil-Red-O, and Masson staining were performed on the sections to determine lesion area and collagen fiber content. For immunohistochemistry staining, after washing with PBS and treatment with 3% H_2_O_2_, sections were blocked at room temperature with 3% bovine serum albumin (Beyotime) for 60 min. Then, sections were incubated with primary antibody at 4°C overnight. On the following day, sections were incubated with secondary antibody at 37°C for 1 h. Finally sections were stained with 3,3N-Diaminobenzidine Tertrahydrochloride (Beyotime). Images were taken by a microscope (Mshot) and analyzed by the Image-pro plus 6.0.

### Statistical Analysis

Statistical analysis was performed using GraphPad Prism 8, when comparing two unpaired groups, if either of the two groups of data did not pass normality test, Mann–Whitley test was used. If the two groups of data passed normality test and the *P*-value of F test ≥ 0.05, two-tailed unpaired Student's *t*-test was used. If the two groups of data passed normality test, but the *P*-value of F test < 0.05, two-tailed unpaired Student's *t*-test with Welch's correction was used. When comparing three or more unpaired groups, if one of the groups of data did not pass normality test, Dunn's multiple comparisons test was used. If all the groups of data passed normality test and had equal square deviation, Tukey's multiple comparisons test was used. If all the groups of data passed normality test but didn't have equal square deviation, Dunnett's T3 multiple comparisons test was used. In all experiments, *P* < 0.05 was considered to be statistically significant. Data are expressed as mean ± SEM. The number of asterisks represent the following: ^*^P < 0.05, ^**^P < 0.01, ^***^P < 0.001.

## Results

### The Activity of PP2A Was Decreased in Human Coronary Atherosclerosis

**Figure 1:**
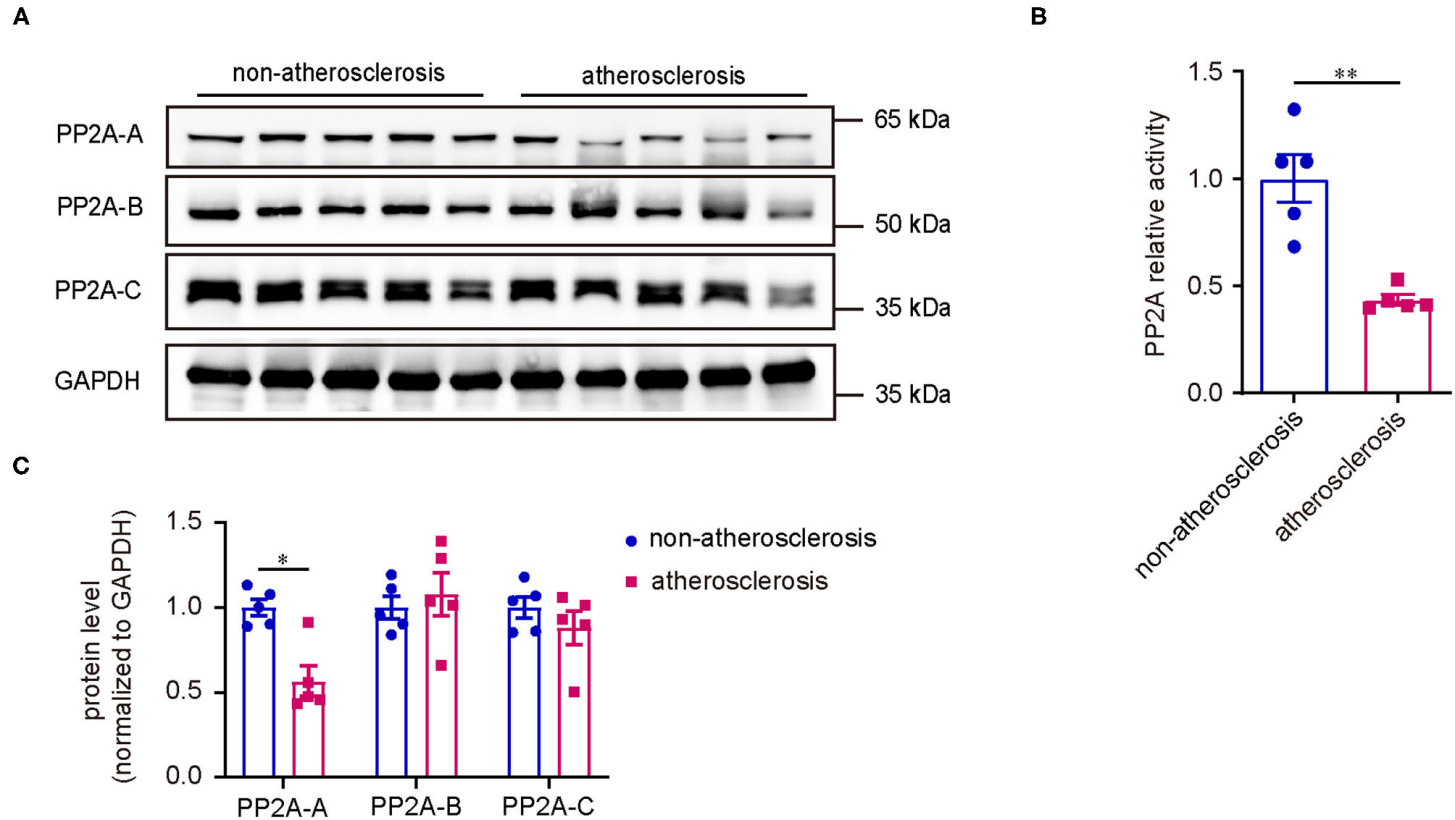
Decreased PP2A expression and activity in human coronary atherosclerosis. **(A)** Upper panel: western blot assessment of PP2A subunits (*n* = 5). Lower panel: western blot quantification. **(B)** PP2A activity assay in the same set of specimens as in **(A)**.

First, to investigate if the expression of PP2A is altered in human coronary atherosclerosis, we analyzed the protein levels of various PP2A subunits in atherosclerotic and non-atherosclerotic human coronary artery specimens by western blot. Compared to non-atherosclerotic arteries, there was an obvious reduction in the expression of PP2A-A subunit in atherosclerotic specimens, whereas the expressions of PP2A-B and PP2A-C subunits had no statistical differences (Figure 1A). Next, to determine whether the deficiency of PP2A-A subunit affects the activity of PP2A, a PP2A immunoprecipitation phosphatase assay kit was used to test the PP2A activity in specimens. Results showed that the activity of PP2A decreased significantly in atherosclerotic arteries when compared to non-atherosclerotic arteries (Figure 1B). Taken together, these findings demonstrate that PP2A is relevant in human coronary atherosclerosis and the function of PP2A is decreased in atherosclerotic human coronary arteries.

### Pharmacological Inhibition of PP2A Aggravated Atherosclerosis in ApoE^–/-^ Mice

Based on the detection of human coronary artery specimens, we hypothesized that PP2A activity inhibition leads to the progression of atherosclerosis. To test our hypothesis, we performed studies using a well-established ApoE^−/−^ atherosclerosis mouse model and a well-characterized hydrophilic small molecule inhibitor of the PP2A, LB100. The safety and tolerability of LB100 has been tested in a phase I clinical trial for the treatment of patients with relapsed solid tumors (21).

**Figure 2:**
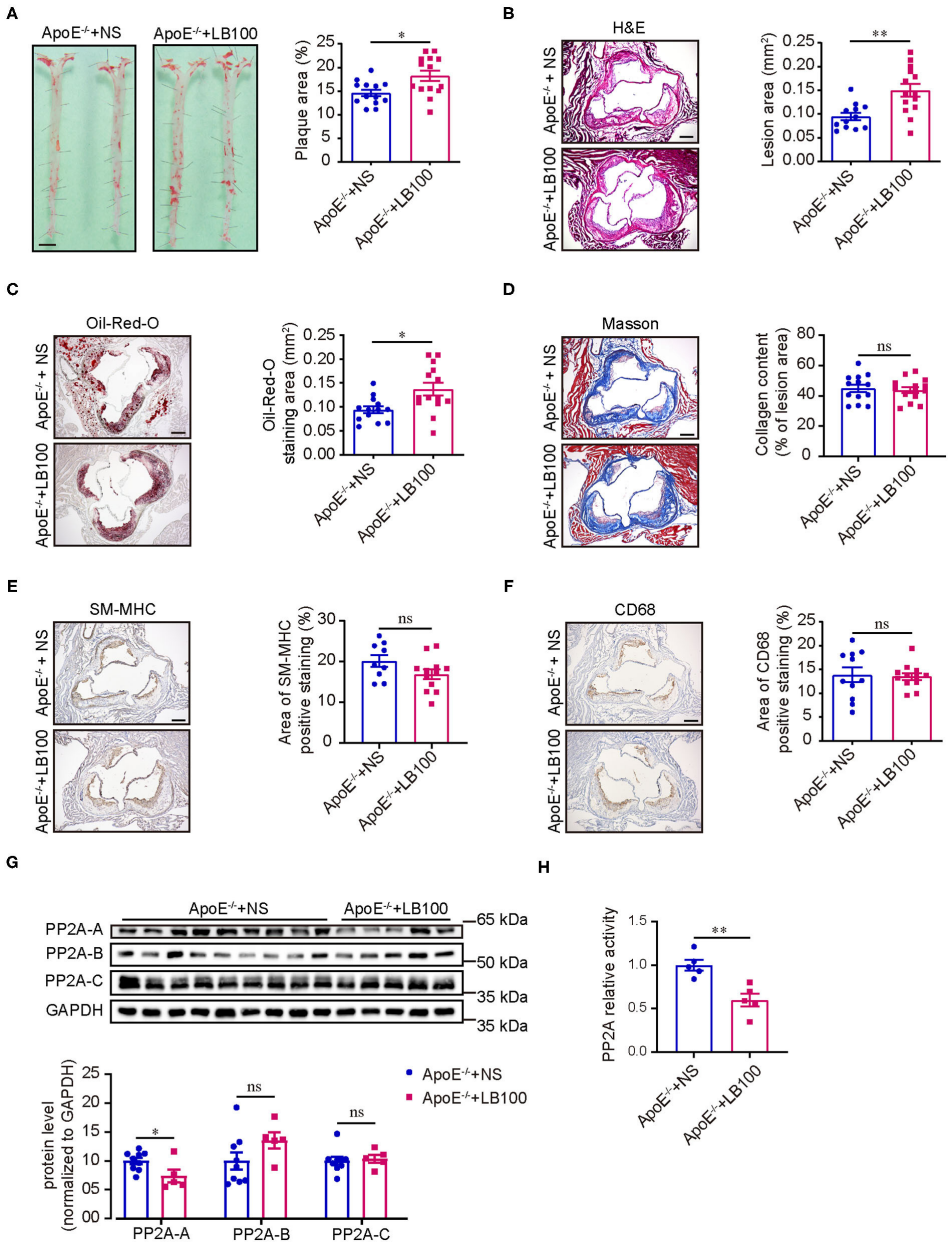
LB100 increased the area of atherosclerotic plaque in ApoE^−/−^ mice. **(A)** Left panel: representative images of whole aortas stained with Oil-Red-O from ApoE^−/−^ mice with NS or LB100 treatment and 12 weeks of western diet feeding. Bar = 2.5 mm. Right panel: quantification of aortic atherosclerotic plaque area (*n* = 13–14). **(B)** Left panel: representative image of aortic root cross sections stained with H & E. Bar = 100 μm. Right panel: quantification of lesion area in aortic root n = 12–14). **(C)** Left panel: representative image of aortic root cross sections stained with Oil-Red-O. Bar = 100 μm. Right panel: quantification of Oil-Red-O staining area in aortic root (*n* = 13–14). **(D)** Left panel: representative image of aortic root cross sections stained with Masson. Bar = 100 μm. Right panel: quantification of collegen fiber content in lesion area (*n* = 13–14). **(E)** Left panel: representative image of SM-MHC immunohistochemistry staining in aortic root cross sections. Bar = 100 μm. Right panel: quantification of SM-MHC positive area (n = 9–12). **(F)** Left panel: Representative image of CD68 immunohistochemistry staining in aortic root cross sections. Bar = 100 μm. Right panel: quantification of CD68 positive area (*n* = 11–12). **(G)** Left panel: western blot assessment of the expression of PP2A subunits in aortas. Right panel: quantification of western blot (*n* = 9–5). **(H)** PP2A activity assay in aortas (*n* = 5).

ApoE^−/−^ mice were treated with normal saline (NS) or LB100 every 2 days and fed a western diet for 12 weeks with body weights measured weekly. After 12 weeks of western diet feeding, mice treated with LB100 displayed significantly increased areas of atherosclerotic plaque in whole aortas compared with the control (Figure 2A). The characteristics of the atherosclerotic plaque were analyzed by H&E staining, Oil-Red-O staining, Masson staining, CD68 immunohistochemistry staining and SM-MHC immunohistochemistry staining for aortic root cross sections. Results showed that LB100 treatment markedly increased the lesion area and lipid deposition (Figures 2B,C). However, there was no significant difference in the content of collagen fiber, the number of smooth muscle cells and the degree of macrophage infiltration in the lesion area between the two groups (Figures 2D–F). To investigate the inhibition effect of LB100, we analyzed the expression levels of PP2A subunits and the PP2A activity in aortas. Compared with aortas from NS-treated ApoE^−/−^ mice, a significant decrease in the expression level of the PP2A-A subunit and the activity of PP2A were observed in aortas of LB-100-treated ApoE^−/−^ mice (Figures 2G,H), which suggests that PP2A was effectively suppressed by LB100 in aortas. No significant differences of the body weights between the two groups of mice were observed during the process of western diet feeding (Supplementary Figure 1A). Finally, we tested the fasting blood total cholesterol and triglyceride levels of ApoE^−/−^ mice with NS or LB100 treatment and 12 weeks of western diet feeding. Once again, no significant differences were found (Supplementary Figures 1B,C). In conclusion, these results indicate that PP2A activity inhibition increases the deposition of lipids and aggravates atherosclerosis in ApoE^−/−^ mice.

### Conditional Knockout of PP2A-Cα in Myeloid Cells Aggravated Atherosclerosis in Mice

Previous studies have shown that PP2A is involved in atherosclerosis by regulating vascular endothelial cells injury, transformation of macrophages and vascular smooth muscle cells to foam cells, and migration of smooth muscle cells (22–26). However, all these studies were limited to *in vitro* cell experiments, and no direct animal experiments showed that PP2A was involved in the occurrence and development of atherosclerosis. PP2A systemic knockout mice are embryonically lethal, and PP2A konckout in cells also results in cell death. Both PP2A-A and PP2A-C subunits have two isoforms. Considering that PP2A-C subunit is a catalytic subunit and has multiple modification sites of amino acid, we decided to construct a conditional knockout mouse of the PP2A-Cα isoform to study the function of PP2A. Since macrophage plays a crucial role in the pathogenesis of atherosclerosis and LB100 treated experiments suggested that inhibition of PP2A activity mainly affected lipids deposition, we decided to produce mice with conditional knockout of the PP2A-Cα subunit in myeloid cells by crossbreeding Cre^Lyz2^ and Ppp2cα^flox/flox^ (fl/fl) mice to investigate whether PP2A deficiency in myeloid cells accelerates the progression of atherosclerosis. Due to the time-consuming and extremely difficult process to obtain Ppp2cα^flox/flox^ Cre^Lyz2^ (cKO) mice on an ApoE^−/−^ or LDLR^−/−^ (low-density lipoprotein receptor knockout) background by crossbreeding cKO mice and ApoE^−/−^ or LDLR^−/−^ mice, we decided to apply in our study a novel, time-saving and effective animal model of atherosclerosis that has been reported and extensively used (27–30).

**Figure 3:**
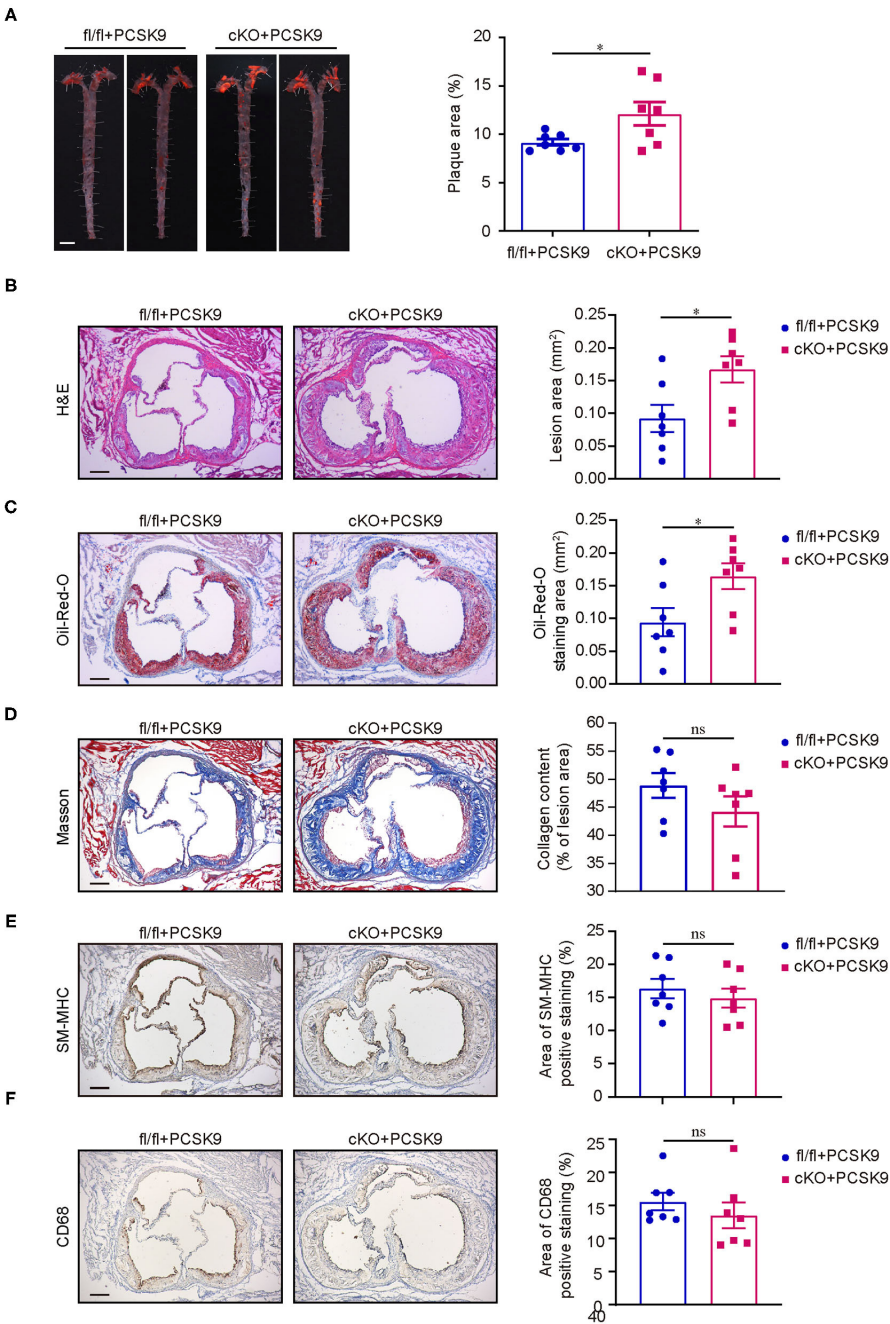
PP2A deficiency in myeloid cells aggravated atherosclerosis in mice. **(A)** Left panel: representative images of Oil-Red-O staining of whole aortas from Ppp2cα^flox/flox^ and Lyz2-Cre/Ppp2cα^flox/flox^ mice injected with AAV-PCSK9 after 16 weeks of high-fat and high-cholesterol diet feeding. Bar = 2.5 mm. Right panel: quantification of aortic atherosclerotic plaque area (*n* = 7). **(B)** Left panel: representative image of aortic root cross sections stained with H & E. Bar = 100 μm. Right panel: quantification of lesion area in aortic root (*n* = 7). **(C)** Left panel: representative image of aortic root cross sections stained with Oil-Red-O. Bar = 100 μm. Right panel: quantification of Oil-Red-O staining area in aortic root (*n* = 7). **(D)** Left panel: representative image of aortic root cross sections stained with Masson. Bar = 100 μm. Right panel: quantification of collegen fiber content in lesion area (*n* = 7). **(E)** Left panel: representative image of SM-MHC immunohistochemistry staining in aortic root cross sections. Bar = 100 μm. Right panel: quantification of SM-MHC positive area (*n* = 7). **(F)** Left panel: representative image of CD68 immunohistochemistry staining in aortic root cross sections. Bar = 100 μm. Right panel: quantification of CD68 positive area (*n* = 7).

Proprotein convertase subtilisin kexin 9 (PCSK9) is a member of the subtilisin serine protease family that can bind to LDL receptors (LDLR) and route them to lysosomes for degradation (31). Single intravenous injection of recombinant adeno-associated viral vector encoding a gain-of-function mutant PCSK9 (AAV-PCSK9) is a rapid and effective method to induce atherosclerosis in mice and a versatile tool for experimental atherosclerosis research (32). On the basis of this method, fl/fl and cKO mice were injected once with AAV-PCSK9 or AAV-GFP (control) *via* caudal vein and then fed with high-fat and high-cholesterol diet. After 16 weeks of high-fat and high-cholesterol diet feeding, mice injected with AAV-PCSK9 had a significant decrease in the expression of LDLR in the liver when compared with the control (Supplementary Figure 2A). Through Oil-Red-O staining of aortas, we found that cKO mice injected with AAV-PCSK9 had notably larger areas of plaques than fl/fl mice injected with AAV-PCSK9 (Figure 3A), and plaques were barely visible in both cKO and fl/fl mice treated with AAV-GFP (Supplementary Figure 2B). Consistent with the results of the aortas, H&E and Oil-Red-Ostaining for aortic root cross sections showed that lesion area and lipid deposition increased in aortic root of cKO mice (Figures 3B,C). However, there was no statistical difference in the content of collagen fiber, the number of smooth muscle cells and the degree of macrophage infiltration in the lesion area of the aortic root between the two groups (Figures 3D–F). Although the aortic lipid deposition increased in cKO mice, there were no obvious differences in body weight between the cKO and fl/fl groups during the progress of high-fat and high-cholesterol diet feeding (Figure 3C). Additionally, no obvious differences were found in fasting blood total cholesterol and triglyceride levels after 16 weeks of high-fat and high-cholesterol diet feeding (Figures 3D,E). Therefore, these data strongly suggest that PP2A deficiency in myeloid cells aggravates atherosclerosis in mice.

### PP2A Deficiency in Macrophages Increased Lipid Uptake and Foam Cell Formation

**Figure 4:**
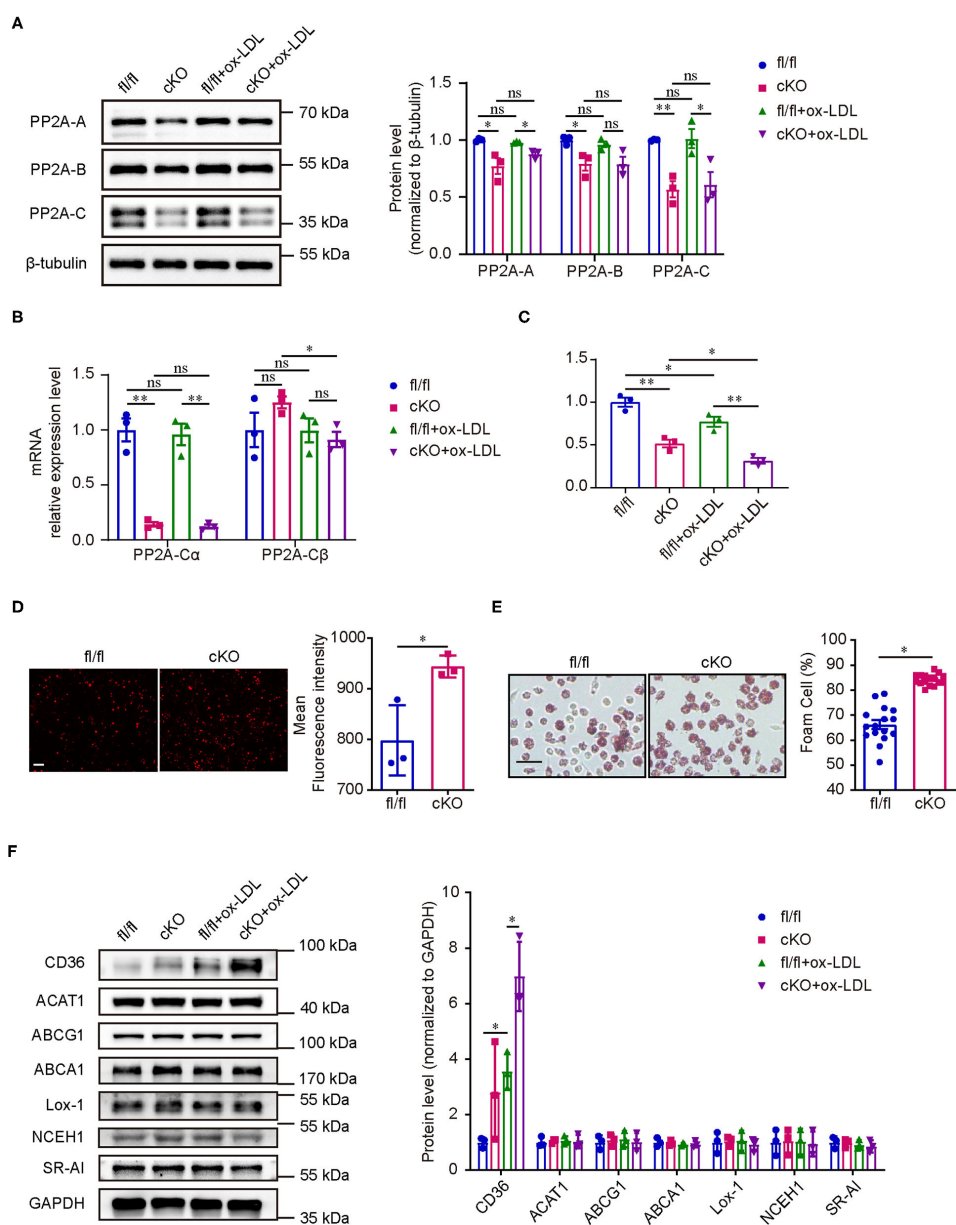
PP2A deficiency in macrophages promoted foam cell formation. **(A)** Left panel: western blot of PP2A subunits in cKO and fl/fl macrophages treated with and without oxLDL for 24 h. Right panel: quantification of western blot (*n* = 3). **(B)** mRNA levels of PP2A-Cα and PP2A-Cβ isoforms in cKO and fl/fl macrophages treated with and without oxLDL for 24 h (*n* = 3). **(C)** PP2A activity assay in cKO and fl/fl macrophages treated with and without oxLDL for 24 h (*n* = 3). **(D)** Left panel: representative images of Dil-oxLDL uptake in peritoneal macrophages isolated from cKO and fl/fl mice. Bar = 100 μm. Right panel: quantification of mean fluorescence intensity (*n* = 3). **(E)** Left panel: foam cell formation in cKO and fl/fl peritoneal macrophages after incubation with oxLDL for 24 h (The experiment was repeated three times, and one of them is shown here). Bar = 100 μm. Right panel: quantification of foam cell formation (16 sights of each group were selected to conduct the quantitative analysis). **(F)** Left panel: western blot of cholesterol homeostasis-related proteins in cKO and fl/fl macrophages treated with and without oxLDL for 24 h. Right panel: quantification of western blot (*n* = 3).

Since PP2A deficiency in myeloid cells aggravated atherosclerosis in mice, we next performed *in vitro* experiments to investigate whether the function of macrophages is regulated by PP2A. Initially, we assessed whether the deficiency of PP2A in macrophages could influence the formation of foam cells, a crucial step in atherosclerosis. Firstly, we tested the effect of PP2A-Cα knockout and oxLDL treatment on the expression and activity of PP2A in macrophages. Peritoneal macrophages from cKO or fl/fl mice were treated with oxLDL or not for 24 h. In cKO macrophages, protein levels of PP2A-A, PP2A-B, and PP2A-C subunits, mRNA level of PP2A-Cα isoform, and the activity of PP2A were markedly decreased (Figures 4A–C), which means the function of PP2A is significantly inhibited. OxLDL treatment had no effect on protein levels of PP2A-A, PP2A-B, and PP2A-C subunits and mRNA level of PP2A-Cα isoform, but the activity of PP2A was significantly decreased (Figures 4A–C). Then, cKO and fl/fl macrophages were incubated with Dil-oxLDL for 4 h, we found that the uptake of oxLDL was significantly increased in cKO macrophages (Figure 4D). Consistent with this, increased foam cell formation was also observed in cKO macrophages when compared to fl/fl macrophages (Figure 4E, Supplementary Figure 3). These *in vitro* observations are in agreement with the increased plaque formation seen in the *in vivo* experiments.

Cholesterol homeostasis is regulated by macrophages through balancing oxLDL uptake, degradation and efflux. To investigate which processes are disturbed by the deficiency of PP2A with an eventual increase in foam cell formation, the expression levels of several key proteins involved in cholesterol homeostasis were analyzed. First, we measured the expression of three major scavenger receptors responsible for oxLDL uptake (CD36, Lox-1, and SR-A1). Interestingly, PP2A deficiency caused a significant increase of the expression of CD36 in oxLDL treated macrophages (Figure 4F). No significant differences were observed in the protein levels of two enzymes involved in the esterification of cholesterol and the hydrolysis of cholesterol ester (ACAT1 and NCEH1) (Figure 4F). Similarly, PP2A deficiency did not alter the expression of ABCA1 and ABCG1, two membrane proteins responsible for cholesterol efflux (Figure 4F). In summary, these findings demonstrated that oxLDL uptake and foam cell formation are disturbed by the increased expression of scavenger receptor CD36 in PP2A-deficient macrophages.

### PP2A Regulated the Expression of CD36 and Foam Cell Formation Through p38 MAPK Signaling Pathway

**Figure 5:**
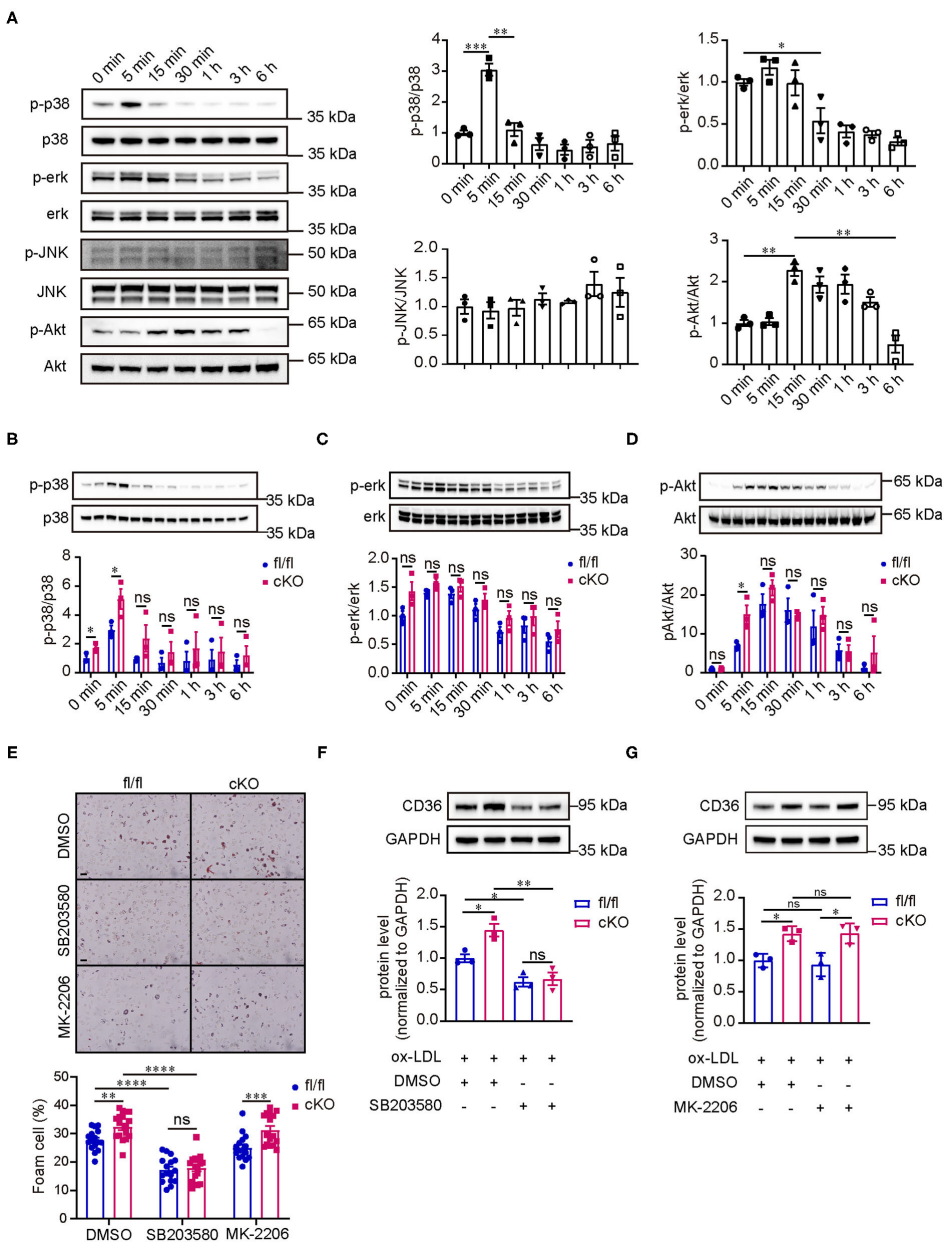
PP2A regulates the expression of CD36 and foam cell formation *via* p38 MAPK signaling pathway. **(A)** Western blot analysis revealed the phosphorylation level of p38/ERK/JNK MAPKs and AKT signaling pathways in peritoneal macrophages isolated from C57BL6 mice treated with oxLDL at 0, 5 min, 15 min, 30 min, 1 h, 3 h, and 6 h (*n* = 3). **(B–D)** Images of western blot detecting the phosphorylation level of relevant signaling pathways at indicated times for cKO and fl/fl peritoneal macrophages after incubation with oxLDL (*n* = 3). **(E)** Upper panel: foam cell formation in cKO and fl/fl peritoneal macrophages treated with inhibitors of signaling pathways and oxLDL (The experiment was repeated three times, and one of them is shown here). Bar = 100 μm. Lower panel: quantification of foam cell formation (16 sights of each group were selected to conduct the quantitative analysis). **(F,G)** Western blot analysis of CD36 in cKO and fl/fl peritoneal macrophages treated with inhibitors of signaling pathways and oxLDL (*n* = 3).

Considering that PP2A is a phosphatase, we studied the changes of signaling pathway phosphorylation in the formation of foam cells. Peritoneal macrophages isolated from C57BL/6 mice were treated with oxLDL for several durations ranging from 0 to 6 h (0, 5 min, 15 min, 30 min, 1 h, 3 h, and 6 h). Western blot analysis showed that p38 and Akt were activated rapidly, reaching their peaks at 5 and 15 min respectively, and then their activity gradually decreased over time (Figure 4A). The activity of ERK was inhibited at 30 min and the decreased activity lasted up to 6 h. The phosphorylation of JNK, however, did not show an obvious changes. Next, we treated peritoneal macrophages from fl/fl and cKO mice with oxLDL for different times to test which signaling pathways are regulated by PP2A. Interestingly, we found that the phosphorylation level of p38 was significantly enhanced in cKO macrophages when compared to fl/fl macrophages at 0 min and 5 min and the phosphorylation level of Akt was also markedly enhanced at 5 min (Figures 4B,E). There was no statistical difference in the activity of ERK between fl/fl and cKO macrophages at each time point analyzed. To further investigate which signaling pathways regulate the expression of CD36 and affect the formation of foam cells, fl/fl and cKO macrophages were treated with SB203580 (p38 inhibitor) or MK2206 (Akt1/2/3 inhibitor) for 4 h, and then treated with oxLDL and the inhibitor for 24 h. Oil-Red-O staining and western blot analysis showed that foam cell formation and expression of CD36 were significantly reduced by the p38 inhibitor, with no significant changes found in the Akt inhibitor treated groups (Figures 5E–G, Supplementary Figure 4). These results revealed that PP2A regulates the expression of CD36 by mediating the activation of the p38 MAPK signaling pathway, thereby affecting foam cell formation.

## Discussion

In this study, we demonstrated that PP2A activity in macrophages is a crucial regulator of foam cell formation and atherosclerosis due to its ability to control the expression of scavenger receptor CD36 through the p38 MAPK signaling pathway.

First, we compared the expression of PP2A subunits and the activity of PP2A holoenzyme between non-atherosclerotic and atherosclerotic human coronary artery specimens by western blot and PP2A immunoprecipitation phosphatase assay kit. To our knowledge, this is the first report to show that the expression of PP2A-A subunit and the activity of the PP2A holoenzyme are significantly decreased in human atherosclerotic arteries. From this data, we hypothesized that the deficiency of PP2A function promotes atherosclerosis. Consistent with our hypothesis, we found that LB100, a inhibitor of PP2A, aggravated the aortic atherosclerosis of ApoE^−/−^ mice *in vivo*. These results indicate that restoring PP2A activity might be a promising strategy for the treatment of atherosclerosis.

It is well established that the occurrence and development of atherosclerosis is a pathological process involving multiple cells in which macrophage foam cell formation is a central link (33). We produced mice with conditional knockout of the PP2A-Cα subunit in myeloid cells to study the effect of PP2A function deficiency in myeloid cells on atherosclerosis. As expected, PP2A deficiency in myeloid cells aggravated the degree of aortic atherosclerosis in mice. *In vitro* studies using peritoneal macrophages indicated that oxLDL uptake and foam cell formation were increased in PP2A-deficient macrophages. Previous studies have shown that PP2A activity is associated with a variety of cellular functions involved in the development of atherosclerosis. In vascular endothelial cells, Yun et al. found that proatherosclerotic transcription factor Yap is dephosphorylated and activated by PP2A, which promotes the development of atherosclerosis (22). Studies from Chen et al. showed that PP2A deactivates eNOS by removing S1177 residue phosphorylation and reduces NO bioavailability (23). Zhang et al. reported that PP2A activation using a PP2A activator DT-061 reduced the uptake and accumulation of lipids in mouse aortic vascular smooth muscle cells (24). Campbell et al. and Yang found that decreased PP2A activity enhances the migration of vascular smooth muscle cells (25, 34). However, in these studies, no cell type-specific PP2A knockout mice were used to directly prove the relationship between PP2A and atherosclerosis. To further pinpoint the role of PP2A in these cells, cell type-specific PP2A knockout mice are required. Future studies are also warranted to determine the roles of other myeloid cells, such as neutrophils and dendritic cells.

Next, our mechanistic experiments showed that increased expression of scavenger receptor CD36 in PP2A-deficient macrophages resulted in enhanced uptake of oxLDL and subsequent foam cell formation. In a previous study, Chen et al. found that inhibiting PP2A activity increased LOX-1 expression, leading to enhanced oxLDL uptake in macrophages (26), but this phenomenon was not observed in our study using PP2A-Cα subunit knockout macrophages. CD36 is a major scavenger receptor for oxLDL, and the binding of oxLDL to CD36 can increase the expression of CD36 by activating peroxisome proliferator-associated receptor-γ (PPAR-γ), the transcription factor of CD36 (35–37). Studies have shown that PPAR-γ activation caused by oxLDL is related to protein kinase B (PKB/Akt) and mitogen-activated protein kinase (MAPK) signaling (38–41). In the present study, we observed that p38 and Akt were activated immediately when macrophages were treated with oxLDL. Moreover, the phosphorylation level of p38 was markedly enhanced in PP2A-deficient macrophages without any intervention. Furthermore, SB203580 (p38 inhibitor), and MK2206 (Akt inhibitor) were used to investigate which signaling pathways affected by PP2A mediate the expression of CD36. The results showed that only the p38 inhibitor could reverse the increase in CD36 expression and foam cell formation in PP2A-deficient macrophages, suggesting that PP2A regulates CD36 expression through the p38 signaling pathway.

In conclusion, our analysis of human atherosclerotic coronary artery specimens and animal experiment using LB100 inhibitor suggested that PP2A plays an important role in the pathogenesis of atherosclerosis. Data from the PP2A-deficient mice and macrophages revealed that PP2A regulates the uptake of oxLDL in marophages through PP2A-p38-CD36 axis.

## Data Availability Statement

The original contributions presented in the study are included in the article/Supplementary Material, further inquiries can be directed to the corresponding author/s.

## Ethics Statement

The studies involving human participants were reviewed and approved by Ethics Committee of Union Hospital, Tongji Medical College, Huazhong University of Science and Technology. The patients/participants provided their written informed consent to participate in this study. The animal study was reviewed and approved by Animal Research Committee of Tongji Medical College, Huazhong University of Science and Technology.

## Author Contributions

CZ, XD, ZL, and ND conceived and designed the study. RL, CZ, and FX performed the experiments, analyzed the data and wrote the manuscript. XZ, XH, and JS contributed to the discussion of the study and the manuscript. All authors read and approved the manuscript.

## Funding

This work was financially supported by the grant from the National Natural Science Foundation of China [81600354 to CZ] and part of the grant from the National Key R and D Program of China [2016YFA0101100 to ND].

## Conflict of Interest

The authors declare that the research was conducted in the absence of any commercial or financial relationships that could be construed as a potential conflict of interest.

## Publisher's Note

All claims expressed in this article are solely those of the authors and do not necessarily represent those of their affiliated organizations, or those of the publisher, the editors and the reviewers. Any product that may be evaluated in this article, or claim that may be made by its manufacturer, is not guaranteed or endorsed by the publisher.

## Supplementary Material

Supplementary Figure 1 **(A)** The changes of body weights during the process of western diet feeding (*n* = 13–14) in NS and LB100 treated mice. **(B,C)** Fasting blood total cholesterol and triglyceride levels after 12 weeks of western diet feeding (*n* = 9–13).

Supplementary Figure 2 **(A)** Western blot assessment of LDLR in the liver of fl/fl and cKO mice injected with AAV-GFP or AAV-PCSK9 after 16 weeks of high-fat and high-cholesterol diet feeding. Right panel: quantification of western blot. **(B)** Images of Oil-Red-O staining of whole aortas from fl/fl and cKO mice injected with AAV-GFP after 16 weeks of high-fat and high-cholesterol diet feeding. Bar = 2.5 mm. **(C)** The change of body weight during the process of high-fat and high-cholesterol diet feeding in cKO and fl/fl mice injected with AAV-GFP (*n* = 7). **(D,E)** Fasting blood total cholesterol and triglyceride levels after 16 weeks of high-fat and high-cholesterol diet feeding (*n* = 7).

Supplementary Figure 3 **(A,B)** Left panel: foam cell formation in cKO and fl/fl peritoneal macrophages after incubation with oxLDL for 24 h. Bar = 100 μm. Right panel: quantification of foam cell formation (16 sights of each group were selected to conduct the quantitative analysis).

Supplementary Figure 4 **(A, B)** Left panel: foam cell formation in cKO and fl/fl peritoneal macrophages treated with inhibitors of signaling pathways and oxLDL. Bar = 100 μm. Right panel: quantification of foam cell formation (16 sights of each group were selected to conduct the quantitative analysis).
