# Supplementary figures and images for: Protein Phosphatase 2A Deficiency in Macrophages Increases Foam Cell Formation and Accelerates Atherosclerotic Lesion Development
Source: Front Cardiovasc Med. 2022 Jan 18;8:745009. doi: 10.3389/fcvm.2021.745009 (PMC8803755; doi:10.3389/fcvm.2021.745009)

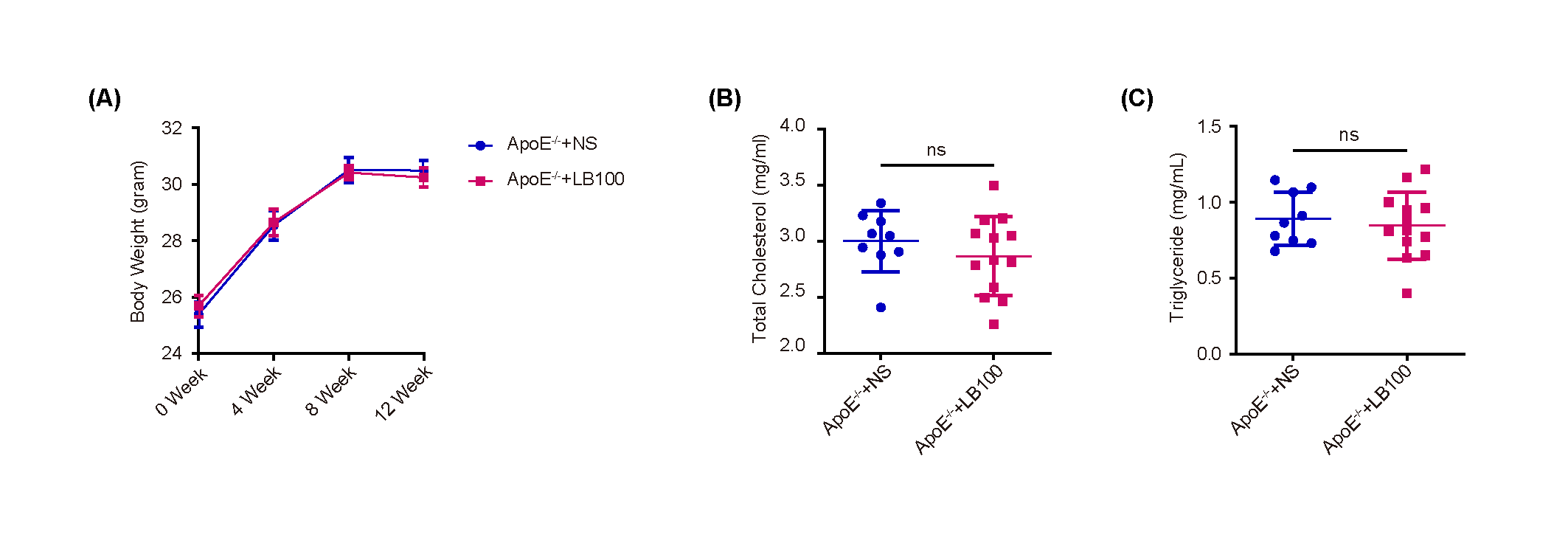

Supplement: Supplementary Figure 1 — (A) The changes of body weights during the process of western diet feeding (n = 13–14) in NS and LB100 treated mice. (B,C) Fasting blood total cholesterol and triglyceride levels after 12 weeks of western diet feeding (n = 9–13). [file Image_1.TIF]

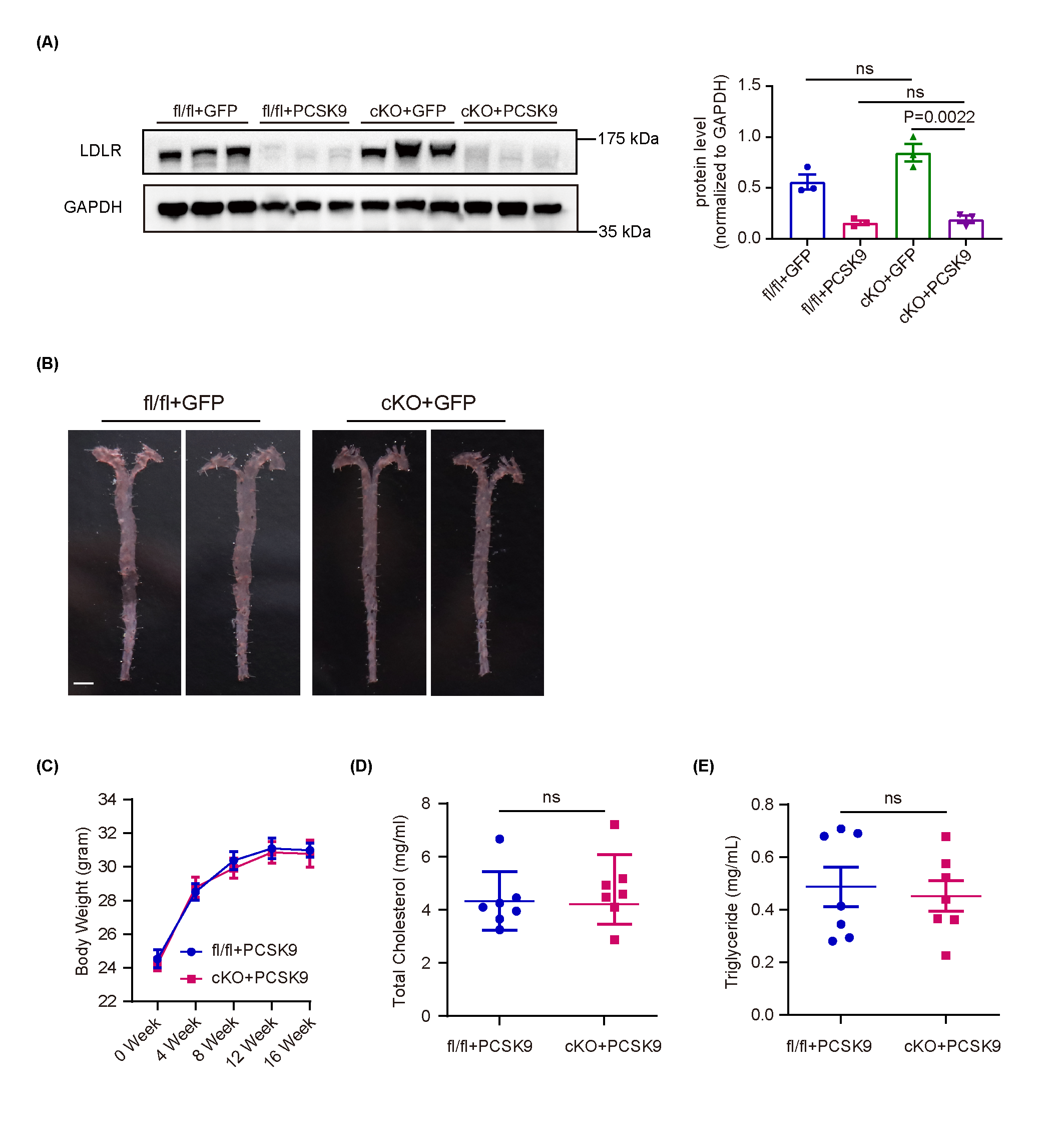

Supplement: Supplementary Figure 2 — (A) Western blot assessment of LDLR in the liver of fl/fl and cKO mice injected with AAV-GFP or AAV-PCSK9 after 16 weeks of high-fat and high-cholesterol diet feeding. Right panel: quantification of western blot. (B) Images of Oil-Red-O staining of whole aortas from fl/fl and cKO mice injected with AAV-GFP after 16 weeks of high-fat and high-cholesterol diet feeding. Bar = 2.5 mm. (C) The change of body weight during the process of high-fat and high-cholesterol diet feeding in cKO and fl/fl mice injected with AAV-GFP (n = 7). (D,E) Fasting blood total cholesterol and triglyceride levels after 16 weeks of high-fat and high-cholesterol diet feeding (n = 7). [file Image_2.TIF]

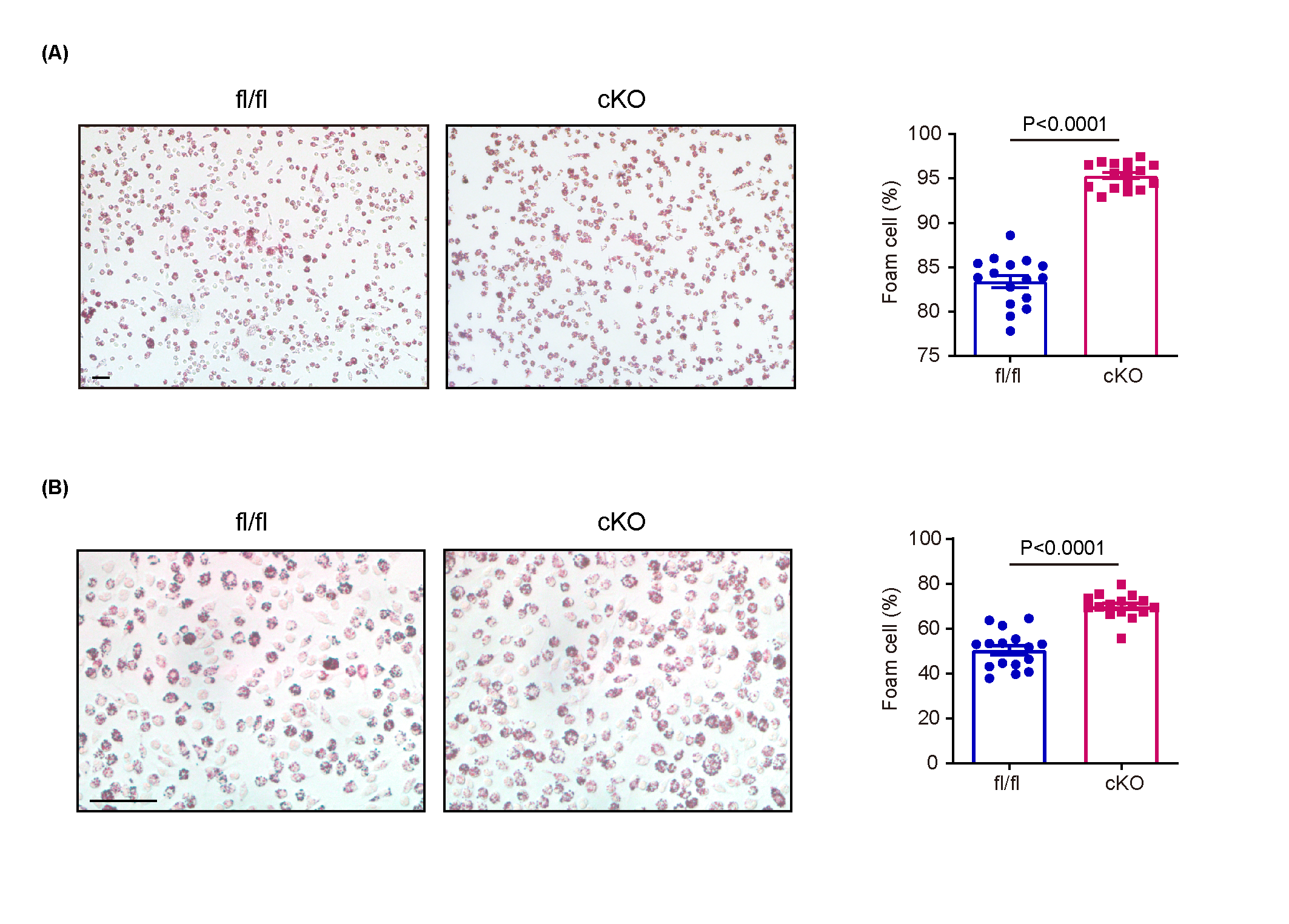

Supplement: Supplementary Figure 3 — (A,B) Left panel: foam cell formation in cKO and fl/fl peritoneal macrophages after incubation with oxLDL for 24 h. Bar = 100 μm. Right panel: quantification of foam cell formation (16 sights of each group were selected to conduct the quantitative analysis). [file Image_3.TIF]

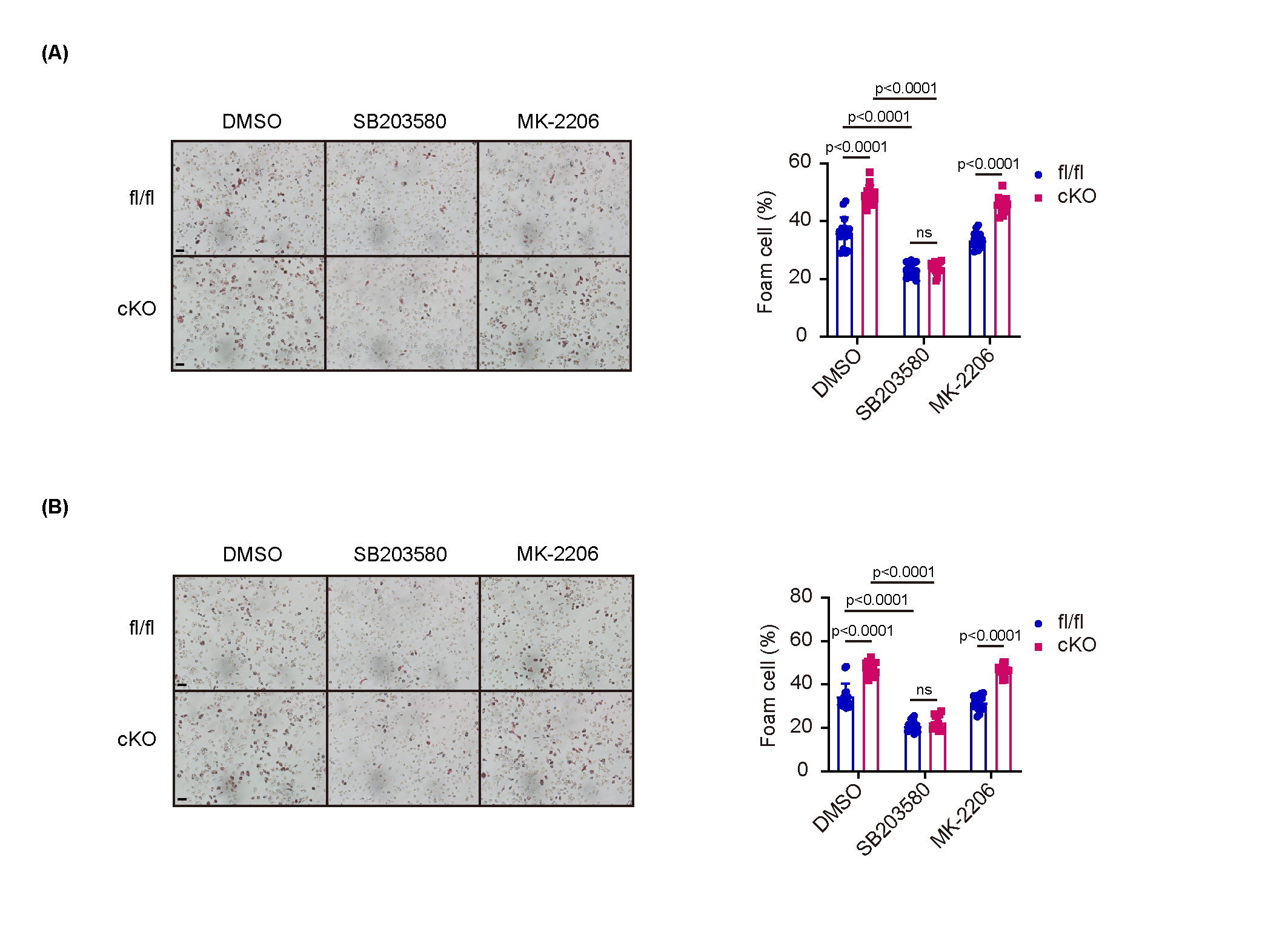

Supplement: Supplementary Figure 4 — (A, B) Left panel: foam cell formation in cKO and fl/fl peritoneal macrophages treated with inhibitors of signaling pathways and oxLDL. Bar = 100 μm. Right panel: quantification of foam cell formation (16 sights of each group were selected to conduct the quantitative analysis). [file Image_4.TIF]
