# Supplementary material for: Protein Phosphatase 2A Deficiency in Macrophages Increases Foam Cell Formation and Accelerates Atherosclerotic Lesion Development
Source: Front Cardiovasc Med. 2022 Jan 18;8:745009. doi: 10.3389/fcvm.2021.745009 (PMC8803755; doi:10.3389/fcvm.2021.745009)
Supplement: Supplementary file 5 [file Table_1.DOCX]

**Supplementary Table 1. Antibodies Used in Western Blotting.**

| **Antibody Name**  **Catalog Number** | **Supplier** | **Species** | **WB dilution** |
| --- | --- | --- | --- |
| PP2A-A  2041S | Cell Signaling  Technology | Rabbit IgG | 1:1000 |
| PP2A-B  4953S | Cell Signaling  Technology | Rabbit IgG | 1:1000 |
| PP2A-C  2038S | Cell Signaling  Technology | Rabbit IgG | 1:1000 |
| GAPDH  60004-1-Ig | Proteintech | Mouse IgG | 1:5000 |
| CD36  MAB2519 | R&D | Rat IgG | 1:1000 |
| ACAT1  NBP1-89285 | NOVUS | Rabbit IgG | 1:1000 |
| ABCG1  ab52617 | abcam | Rabbit IgG | 1:1000 |
| ABCA1  NB100-2068 | NOVUS | Mouse IgG | 1:1000 |
| Lox-1  NB100-2527 | NOVUS | Rabbit IgG | 1:1000 |
| NCEH1  NBP1-79318 | NOVUS | Rabbit IgG | 1:1000 |
| SR-AI  MAB1797 | R&D | Rat IgG | 1:1000 |
| Phospho-p38 (Thr180/Tyr182)  4511S | Cell Signaling  Technology | Rabbit IgG | 1:1000 |
| p38  8690S | Cell Signaling  Technology | Rabbit IgG | 1:1000 |
| Phospho-ERK1/2 (Thr202/Tyr204)  9101S | Cell Signaling  Technology | Rabbit IgG | 1:1000 |
| ERK1/2  4695S | Cell Signaling  Technology | Rabbit IgG | 1:1000 |
| p-SAPK/JNK (Thr183/Tyr185)  9255S | Cell Signaling  Technology | Mouse IgG | 1:1000 |
| SAPK/JNK  9252S | Cell Signaling  Technology | Rabbit IgG | 1:1000 |
| Phospho-Akt (Ser473)  4060S | Cell Signaling  Technology | Rabbit IgG | 1:1000 |
| Akt  4691S | Cell Signaling  Technology | Rabbit IgG | 1:1000 |
| GAPDH  60004-1-Ig | Proteintech | Mouse IgG | 1:5000 |
| Anti mouse IgG-HRP  7076 | Cell Signaling  Technology |  | 1:3000 |
| Anti rabbit IgG-HRP  7074 | Cell Signaling  Technology |  | 1:3000 |
| Goat Anti-Rat IgG(H+L)  HRP conjugate  SA00001-15 | Proteintech |  | 1:5000 |
