# Supplementary material for: Protein Phosphatase 2A Deficiency in Macrophages Increases Foam Cell Formation and Accelerates Atherosclerotic Lesion Development
Source: Front Cardiovasc Med. 2022 Jan 18;8:745009. doi: 10.3389/fcvm.2021.745009 (PMC8803755; doi:10.3389/fcvm.2021.745009)
Supplement: Supplementary file 6 [file Table_2.DOCX]

**Supplementary Table 2. Primers Used in Quantitative PCR.**

| **Gene Name** | **Primer Sequence** |
| --- | --- |
| Mus PPP2CA | F: 5’ – TCGGGGAGCTGGTTATACCT – 3’;  R: 5’ – GCAGCTTGGTTACCACAACG – 3’ |
| Mus PPP2CB | F: 5’ – CTGAACGAGAACCAAGTGCG – 3’;  R: 5’ – CCACAGACGGTAACAGGACA – 3’ |
| Mus Gapdh | F: 5’ – CCTCGTCCCGTAGACAAAATG – 3’;  R: 5’ –TGAGGTCAATGAAGGGGTCGT – 3’ |
